# A paper/polymer hybrid microfluidic microplate for rapid quantitative detection of multiple disease biomarkers

**DOI:** 10.1038/srep30474

**Published:** 2016-07-26

**Authors:** Sharma T. Sanjay, Maowei Dou, Jianjun Sun, XiuJun Li

**Affiliations:** 1 Department of Chemistry, University of Texas at El Paso, 500 West University Ave, El Paso, Texas, 79968, USA; 2 Border Biomedical Research Center, University of Texas at El Paso, 500 West University Ave, El Paso, Texas, 79968, USA; 3 Biomedical Engineering, University of Texas at El Paso, 500 West University Ave, El Paso, Texas, 79968, USA

## Abstract

Enzyme linked immunosorbent assay (ELISA) is one of the most widely used laboratory disease diagnosis methods. However, performing ELISA in low-resource settings is limited by long incubation time, large volumes of precious reagents, and well-equipped laboratories. Herein, we developed a simple, miniaturized paper/PMMA (poly(methyl methacrylate)) hybrid microfluidic microplate for low-cost, high throughput, and point-of-care (POC) infectious disease diagnosis. The novel use of porous paper in flow-through microwells facilitates rapid antibody/antigen immobilization and efficient washing, avoiding complicated surface modifications. The top reagent delivery channels can simply transfer reagents to multiple microwells thus avoiding repeated manual pipetting and costly robots. Results of colorimetric ELISA can be observed within an hour by the naked eye. Quantitative analysis was achieved by calculating the brightness of images scanned by an office scanner. Immunoglobulin G (IgG) and Hepatitis B surface Antigen (HBsAg) were quantitatively analyzed with good reliability in human serum samples. Without using any specialized equipment, the limits of detection of 1.6 ng/mL for IgG and 1.3 ng/mL for HBsAg were achieved, which were comparable to commercial ELISA kits using specialized equipment. We envisage that this simple POC hybrid microplate can have broad applications in various bioassays, especially in resource-limited settings.

Acute infectious diseases caused by various pathogenic microorganisms have been the major cause of global death and disability rate throughout the human history, especially in underdeveloped and developing countries^1^. Our defense against these diseases has been facing problems like recognition of the pathogens or viruses’ species, strains, virulence factors, and antimicrobial susceptibilities in a timely manner^2^. These diseases are usually diagnosed by exhausting immunoassay tests like enzyme linked immunosorbent assay (ELISA), immunofluorescence, western blotting, and immunodiffusion along with polymerase chain reaction (PCR), flow cytometry and a wide range of other techniques^3^,^4^,^5^. Although there is a considerable progress in science and technology, rapid and sensitive point-of-care (POC) detection of diseases or biological agents in low-resource settings (e.g. high-poverty regions) is still a challenge^6^. The characteristics of POC devices are summarized in the World Health Organization’s guidelines as ASSURED (Affordable, Sensitive, Specific, User-friendly, Rapid treatment and robust use, Equipment free, and Delivered to those in needs)^7^.

ELISA, one of the most commonly and widely used laboratory methods in medical diagnostic and research applications, detects proteins based on their binding to immobilized antibodies or antigens. Even though most ELISAs today which are performed in 96-well plates are well suited for high throughput assays, they take several hours to complete because of the hour-long incubation and blocking time^8^. Likewise, other critical issues include consumption of large volumes of precious samples and reagents, and dependence on laboratory settings, making conventional ELISA not suitable for POC detection^8^. In addition, highly complicated and specialized instruments have to be utilized to automate assays in a high-throughput format, including robotic pipetters, plate washers, and optical detectors. Furthermore, most detection methods require bulky and expensive equipment, which further limits their application in POC detection and in developing countries.

Microfluidic lab-on-a-chip (LOC) devices, mostly produced by the microfabrication technique, possess astonishing features for low-cost, simple, and rapid bioanalysis. Microfluidic immunoassay devices possess remarkable features such as high surface-to-volume ratio and microliter volumes of microchannels, which leads to significant decrease in analysis time from hours to minutes and minimal reagent consumption as compared to regular ELISA. These highly portable microfluidic devices with integrated processing can analyze complex biological fluids including serum, urine^9^, cells, and cell lysates for various applications, such as detection of diseases^10^,^11^,^12^, single cell analysis^13^,^14^,^15^, 3D cell culture for tissue-based bioassays^16^, forensic analysis^17^, and a wide range of other fields^18^,^19^,^20^,^21^,^22^.

To address issues from conventional microplates, a few microplate-format microfluidic devices have been developed for immunoassays. For instance, Kai *et al*.^8^ developed a 96-well microfluidic microplate for ELISA with improved sensitivity and reduced sample volumes. The microplate was fabricated with clear polystyrene through injection molding. Each well was connected to a microfluidic channel on the opposing face of the substrate via a through reservoir at bottom of the well. The ELISA on this microfluidic microplate took less time and consumed less reagents as compared to conventional ELISA, but it still required a fluorescence microplate reader. Sapsford *et al*.^23^ developed a miniaturized 96-well microfluidic chip for portable ELISAs with colorimetric detection. The 96-well ELISA chip was micro-machined using clear acrylic and polycarbonate (PC) bound together by double sided tape. Although the reagent consumption was less than conventional ELISA and a portable detector (electroluminescence semiconductor strip with a charge coupled device (CCD)) was used, overnight incubation and manual fluid handling was required. Similarly, Sun *et al*.^24^ fabricated a miniaturized 96-well device for immunological detection, assembling six layers of poly(methyl methacrylate (PMMA) core and five PC layers. They performed electrochemiluminescence ELISA of staphylococcal enterotoxin B (SEB) using a CCD detector. The microfluidic device required a complicated functionalization and device assembling steps along with long incubation time to complete the assay. Overall, all these devices require either long incubation time, surface functionalization or complicated detection systems.

With the emergence of paper-based devices in recent years, various POC analyses, including paper-based ELISAs have been developed^25^,^26^,^27^. Paper-based devices, which don’t require clean room for fabrication, can transport liquid via capillary effect and don’t require external force. Another significant feature of paper is the high surface to volume ratio of the micro-porous structure, which improves the immobilization of protein and other biological agents. Paper-based ELISA takes advantage of high specificity of ELISA and low cost, easy-to-use paper-based devices. Whitesides and his colleagues performed ELISA in a 96-microzone plate fabricated in paper^28^. Although it was faster and less expensive, it was less sensitive than conventional ELISA. Murdock *et al*. used 96-well paper-based ELISA for the assay of human performance biomarker^29^. They used complicated and time-consuming conjugation steps to perform enzyme-free ELISA using gold nanoparticles. Wang *et al*. performed chemiluminescence ELISA of tumor markers on a paper-based device (6 × 3 zones). Chitosan coating and glutaraldehyde cross-linking were required to covalently immobilize antibodies to perform bioassay for tumor markers^30^. In addition, Lei *et al*. performed paper-based immunoassay (8 × 6 zones) for detection of influenza^31^. The limitations in paper-based ELISA include low-performance in flow control and the need of repeated micropipetting for adding reagents and washing all the zones, which is really time-consuming and limits its application for high-throughput detection especially in low-resource settings. In addition, we observed that the repeated washing steps in the micro-zones leads to spreading of the reagents over the hydrophobic areas, which is one of the serious problems faced in paper-based devices.

Along with paper, some polymers such as PMMA have also been widely used for the fabrication of microfluidic devices. Each substrate has its own advantages and disadvantages. PMMA is transparent, rigid and rapidly delivers reagents to different regions. However, polymers such as PMMA require complicated surface modification procedures to immobilize biosensors and other biomolecules such as antibodies and enzymes. For instance, ELISA has been reported in PMMA devices but they require complicated surface modifications including poly(ethyleneimine) (PEI) treatment^32^,^33^, (3-aminopropyl)triethoxy silane (APTES) treatment^34^, and carbon nanotube (CNT) functionalization^24^. In addition, they require detectors like fluorescence microscopy^33^,^34^. On the contrary, paper-based devices can rapidly immobilize biosensors and other biomolecules but do not offer high performance in flow control especially over a fairly long distance. Hybrid devices can take advantages of various substrates, while eliminating some limitations of certain substrates. Recently, hybrid devices have been used for various applications. Our group developed a polydimethylsiloxane (PDMS)/paper hybrid microfluidic biochip integrated with aptasensors for one-step multiplexed pathogen detection^35^. Paper in this hybrid device acted as the substrate for facile immobilization of aptamer-functionalized nano-biosensors without complicated surface modification. Recently, Dou *et al*. fabricated another PDMS/paper hybrid microfluidic platform integrated with loop-mediated isothermal amplification for detection of meningitis-causing bacteria^36^. It was interesting that they found the hybrid device provided more stable performance than non-hybrid devices over a period of 2 months. These types of hybrid devices have been used for various applications including infectious diseases diagnosis^35^,^36^,^37^.

Herein, we have developed a simple miniaturized 56-microwell paper/PMMA hybrid microfluidic ELISA microplate for rapid and high-throughput detection of infectious diseases. A series of novel funnel-shaped PMMA microwells have been created by laser ablation of PMMA, wherein a paper substrate can be placed to complete ELISA within an hour. The introduction of 3D micro-porous paper with high surface-to-volume ratio in microwells of this hybrid microplate facilitated rapid immobilization of antibody/antigen and also avoided complicated surface modifications. The top reagent delivery channels along with the vertical flow-through microwells in the middle PMMA layer can simply transfer reagents to multiple microwells, thus avoiding repeated manual pipetting and washing steps into each well in conventional ELISA or the use of costly robots. All the reagents/analytes pass through the 3D matrix of the paper surface from the funnel-shaped microwells. This design not only provides efficient washing, but also increases the opportunities of analytes to be rapidly and efficiently captured, thus resulting in higher detection sensitivity. ELISA of Immunoglobulin G (IgG) and Hepatitis B surface antigen (HBsAg) were performed in the hybrid device and limits of detection (LODs) comparable to commercial ELISA kits were obtained by using an office scanner, without the use of any specialized instruments like a microplate reader.

## Results and Discussions

### Hybrid microfluidic microplates

**Figure 1:**
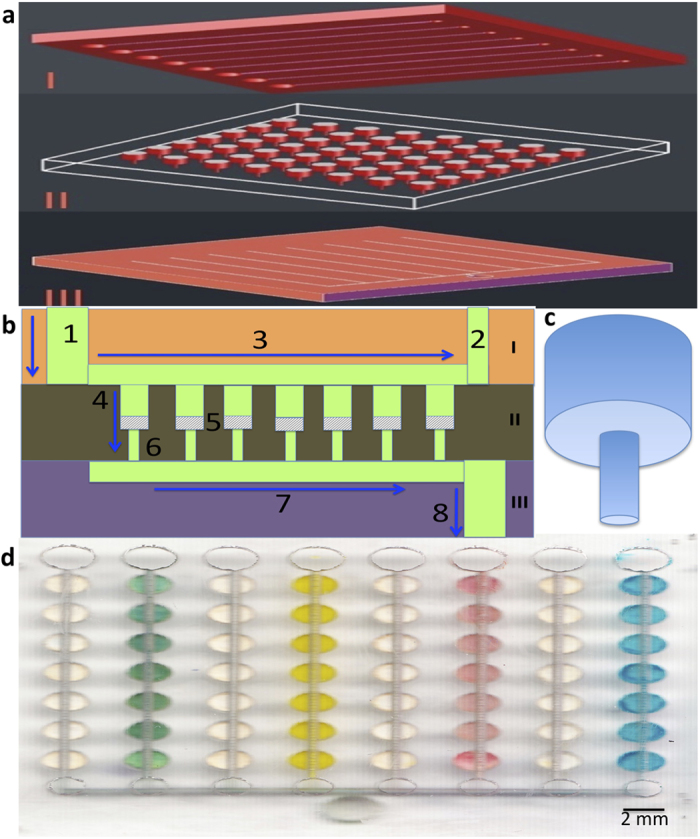
Chip design of the PMMA/paper hybrid microfluidic microplate. (**a**) 3D schematic of the exploded view of the hybrid device. (**b**) Cross-section view of the chip. The chip consists of three PMMA layers. The top layer (I) for fluid delivery consists of inlet reservoir ‘1’ and fluid distribution channel ‘3’. The middle layer (II) for incubation consists of 56 unique funnel-shaped microwells, with upper microwell ‘4’ and lower microwell ‘6’, with paper disks ‘5’ placed in between. The lowermost layer (III) for fluid removal consists of an outlet channel ‘7’, which leads to a common outlet reservoir ‘8’. (**c**) 3D exploded view of the funnel-shaped microwell. (**d**) Photograph of the actual assembled device with water and different colored dyes in alternate columns.

ELISA of multiple disease biomarkers was performed using the hybrid device. As shown in Fig. 1a, the microfluidic device consists of three different layers. The topmost layer, the fluid delivery layer, is used to deliver all the assay reagents and also forms the cover for the microwells in the assay plate (middle layer). Each of the channels connected to different inlet reservoirs of upper layer delivers reagents to 7 microwells in the middle layer. Pieces of chromatography paper were placed inside each microwells. The middle layer contains funnel-shaped (Fig. 1c) microwells with an upper diameter of 2 mm and a lower diameter of 0.3 mm, wherein a paper disk can be placed, as shown in the cross section view of the device from Fig. 1b. The 0.3 mm diameter-lower microwells are placed just below the upper microwells of the middle layer and helps to hold the paper in place and minimize the chances of backflow of the reagents. Just underneath the bottom of the assay microwells, is attached the outlet system. The outlet channels are connected to a single outlet microwell, which acts as an outlet reservoir once a negative pressure is applied. Arrows in Fig. 1b shows the direction of the flow of reagents. Figure 1d shows the photograph of the fully assembled microfluidic chip filled with different food dyes.

Since the funnel-shaped microwells involve different depths, multi-level fabrication is needed. Although photolithography is one of the most widely used fabrication techniques to fabricate microfluidic devices, it is difficult, expensive and complicated to create a microfluidic device with different depths. It requires multi-level microfabrication, alignment and multiple photomasks^38^,^39^,^40^. On the contrary, laser ablation is a rapid prototyping method for the fabrication of microfluidic devices. It uses high intensity laser beams to evaporate polymers at the focal point. The evaporation is due to photo-degradation or thermal-degradation or the combination of both^11^,^41^. By applying different intensities, microstructures with different depths can be readily fabricated. Therefore, we developed a simple laser ablation method to create the funnel-shaped microwells, which can be completed within minutes, without using any photomask (see Supplementary Fig. S1 and Supplementary Information online for more details).

After the assembly of the hybrid microfluidic microplate, cross-contamination test was done as microwells were connected through channels at the bottom layer. For cross-contaminations test, fluorescein isothiocyanate (FITC) was added to the alternate columns of the device, while Milli Q water was added in the adjacent columns. As seen from the fluorescent image (see Supplementary Fig. S2), high fluorescent intensity was only observed in the alternate column (a, c, e, and g) where FITC was added; there was no fluorescence in adjacent columns (b, d, f, and h). The result shows that there was no cross contamination or leakage within different columns. To confirm this, different colored dyes were similarly passed into the alternate columns, with water in the adjacent columns. Similar results were obtained with colours showing up only in the alternate columns and clear background in the adjacent columns (Fig. 1d). This further confirmed that there was no cross-contamination between the adjacent columns.

**Figure 2:**
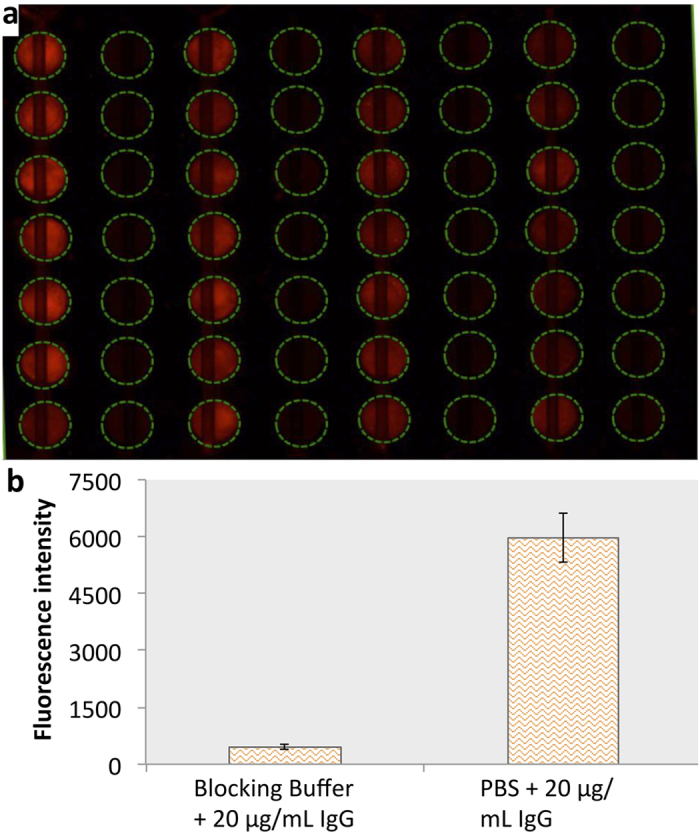
Rapid immobilization of antibodies (**a**) and effectiveness of blocking buffer (**b**) for ELISA on the hybrid device. (**a**) Fluorescence image of Cy3-labeled IgG immobilized on the hybrid device. Different concentrations of IgG includes 100 μg/mL, PBS, 50 μg/mL, PBS, 25 μg/mL, PBS, 12.5 μg/mL, and PBS, respectively, from left to right. (**b**) The mean fluorescence intensity of 20 μg/mL of Cy-3 IgG immobilized on the hybrid device with and without the blocking buffer.

Because of the novel introduction of paper in this hybrid microfluidic microplate, antigen/antibody can be quickly immobilized within 10 minutes as compared to overnight incubation in conventional microplates^28^. Cy3-labeled IgG was used to assess rapid immobilization of antibody on paper surface. Different concentrations of Cy3-labeled IgG (100, 50, 25, and 12.5 μg/mL) were introduced into alternate columns of the device, and PBS in the adjacent columns for 10 minutes. After washing, from Fig. 2a, we can see the decreasing intensity of fluorescence in the alternate columns (from left to right), with the decrease in concentration of IgG. There was no fluorescence in the adjacent columns where PBS was added. Yet, in another experiment the blocking buffer (4% BSA + 0.05% Tween 20) was added to one column and PBS to another to test the effectiveness of the blocking buffer. After 10 minutes, Cy3-labeled IgG was added to both and incubated for 10 more minutes followed by washing three times with PBST. Figure 2b shows that blocking buffer can be used to effectively block the paper surface. Minimal fluorescence can be seen in the column where blocking buffer was added before the addition of Cy3-labeled IgG.

The hybrid microfluidic device has several important features. First, the reagents can be easily transferred to all the microwells, minimizing time-consuming repeated micropipetting to add reagents. PMMA acts as the support for paper and provides channels for reagent delivery. As such, it can overcome the slow flow issue on paper. Flow of reagents can be controlled in a better way with the hybrid device. Second, due to the novel introduction of paper in this hybrid microplate, antigen/antibody can be rapidly immobilized in the paper surface and does not require the complicated surface modification of PMMA. Additionally, due to the flow-through funnel-shaped microwells, the entire reagent passes through the micro-porous paper, which results in not only more efficient and rapid antigen/antibody immobilization, but also more efficient washing. Efficient washing is also very important to decrease the background especially in paper-based ELISA.

### Optimization of incubation time for BCIP/NBT

**Figure 3:**
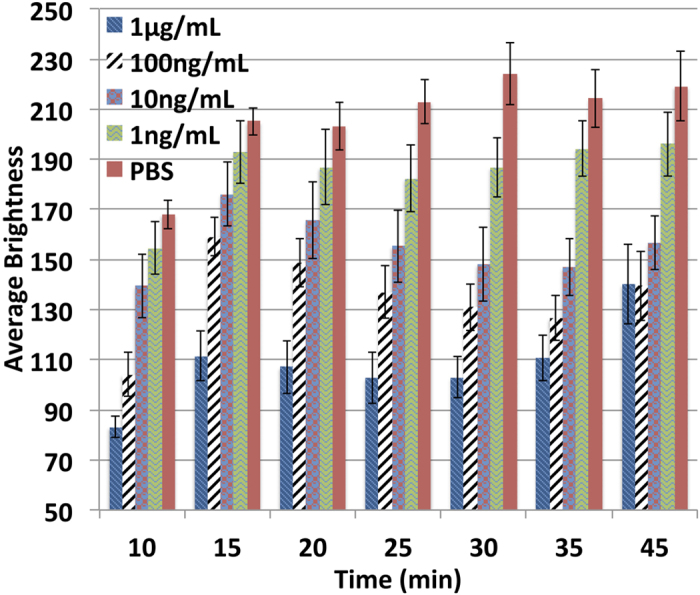
Optimization of the incubation time for BCIP/NBT. The graph shows the average brightness value of the enzymatically-developed colour as measured by ImageJ for different IgG concentrations against the incubation time.

It was observed that once BCIP/NBT was added to the chip, the substrate started to produce an insoluble diformazan end product, which was purple in colour and could be observed visually. As average brightness was measured after the assay, we can observe that the higher concentration of the analyte produces dark purple colour resulting in lower brightness and vice-versa. The colour intensity significantly increased to a certain time and then started fading away. To optimize the optimal incubation, on-chip ELISA of IgG (from 1 ng/mL-1 μg/mL) was performed. The chip was scanned every 5 minutes, starting from 10 minutes after the addition of BCIP/NBT. As seen from Fig. 3, the purple colour started fading away after 30 minutes, which leads to increase in average brightness value. In addition, lower signal/noise ratios (the noise was derived from the column with PBS only) were observed starting at 30 minutes. Colour intensity of 20-minute incubation was almost similar to that of 25-minute incubation, but 20-minute incubation had higher background (lower brightness of PBS). It can also be noticed that the deviation started increasing slightly after 25 minutes. Therefore, considering the signal/noise ratio, deviation, and the time required, 25 minutes incubation time was considered optimum and was used in subsequent experiments.

### Rapid Quantitative Detection of IgG

**Figure 4:**
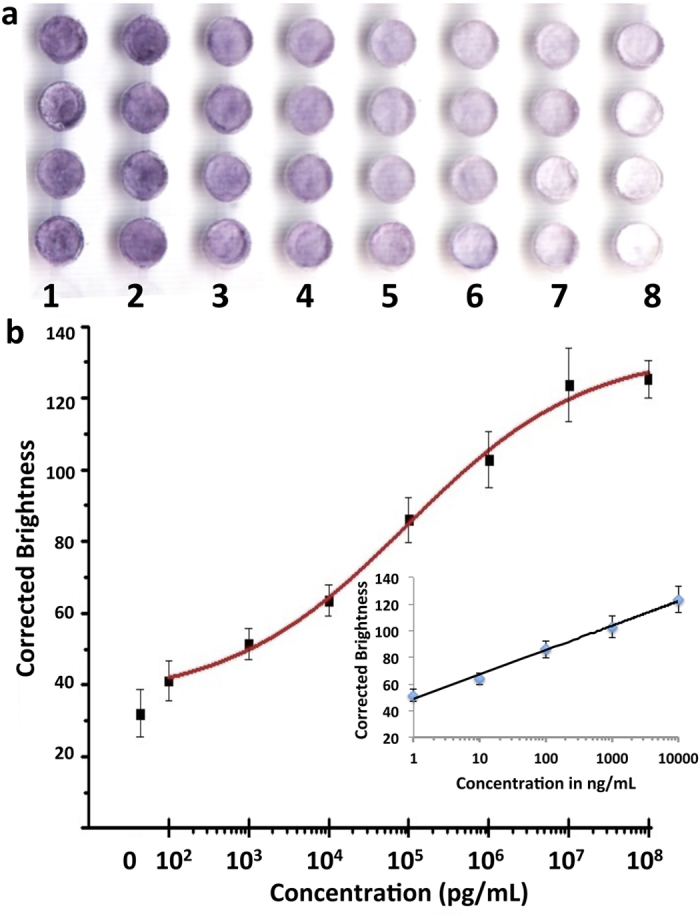
Rapid detection of IgG by on-chip ELISA on a hybrid microfluidic microplate. (**a**) Partial scanned image of the microfluidic microplate with different IgG concentrations by an office scanner: (1) 100 μg/mL, (2) 10 μg/mL, (3) 1 μg/mL, (4) 100 ng/mL, (5) 10 ng/mL, (6) 1 ng/mL, (7) 0.1 ng/mL and (8) 0 ng/mL. (**b**) Sigmoidal plot of the corrected brightness of microwells versus different IgG concentrations. The inset shows the calibration curve of IgG where the range of linearity lies between 1 ng/mL to 1 × 10^4^ ng/mL.

IgG is the most common type of antibody found in the human circulation (75% of serum antibodies). The measurement of IgG can be a diagnostic tool for conditions like autoimmune hepatitis^42^. IgG levels are indicative of immune status of diseases such as measles, mumps, and rubella (MMR), hepatitis B virus, and varicella^43^. In addition, IgG can serve as a specific marker for Neuromyelitis optica, an inflammatory demyelinating disease^44^. Thus, we first demonstrated the application of our hybrid microfluidic plate for rapid detection of IgG (0.1 ng/mL-100 μg/mL). For the on-chip ELISA, all reagents were loaded sequentially from the inlets in the upper layer of the PMMA chip, the reagent delivery system. No external power or device was used for the addition of reagents, except a micropipette. After ELISA was completed, the result could be viewed by the naked eye, or a portable office scanner can be used to scan the device. Figure 4a shows a scanned image for IgG detection from an office scanner. It was found that the colour intensity increased as the IgG concentration increased from 0.1 ng/mL to 100 μg/mL (from right to left) with the blank in the rightmost column. Signal intensities of the scanned images were calculated using ImageJ. Figure 4b shows the calibration curve of IgG over a concentration range of 1 × 10^2^ pg/mL to 1 × 10^8^ pg/mL. A sigmoidal curve (Fig. 4b) was observed over the whole concentration range, while the linearity lies between 1 × 10^3^ pg/mL to 1 × 10^7^ pg/mL (inset Fig. 4b) with a regression curve of y = 18.35 log (x) + 48.74 (r^2^ = 0.99), which illustrates a typical immunoassay characteristic.

The LOD for IgG was calculated to be 1.6 ng/mL based on 3 folds of standard deviation (SD) above the blank value, which was comparable to commercial 96-well microplate ELISA (LOD, 1.6–6.25 ng/mL)^45^. The conventional 96-well microplate ELISA not only consumes more reagents (50–100 μL), and requires overnight incubation, but also rely on specialized instruments like a microplate reader. However, our method only needs 5 μL samples, and 1 h to complete the whole assay, without using any specialized instruments. A more detailed comparison is listed in Table 1. As to PMMA devices, they require complicated surface modification with APTES and long incubation time (i.e. 12 hours), and the LOD was only 0.12 μg/mL even with a fluorescence microscope^34^. Although 96-zone paper-based ELISA did not require surface modification^28^, it required time-consuming repeated micropepitting, making it less user-friendly and incapable of high-throughput detection. Additionally, the LOD of paper-based ELISA was 54 fmol/zone, much higher than that of our hybrid system (53.6 amol/zone), indicating high sensitivity of our method, which might be attributed to efficient washing from our hybrid system.

### Rapid Quantitative Detection of HBsAg

Hepatitis B virus (HBV) infection is a major cause of chronic hepatic damage and of hepatocellular carcinomas worldwide^46^. HBsAg, a serological biomarker for a HBV infection, can diagnose acute and chronic hepatitis B virus^47^,^48^,^49^. The titer of serum HBsAg indicates the level of infection and severity of the disease^49^,^50^.

**Figure 5:**
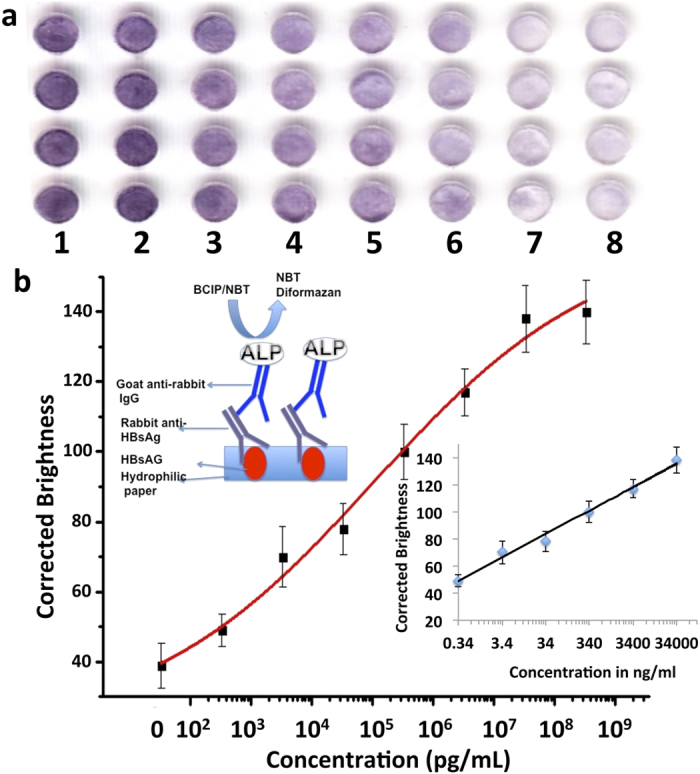
Detection of HBsAg by ELISA on a hybrid microfluidic microplate. (**a**) Partial scanned image of the microfluidic microplate with different HBsAg concentrations by an office scanner: (1) 340 μg/mL, (2) 34 μg/mL, (3) 3.4 μg/mL, (4) 340 ng/mL, (5) 34 ng/mL, (6) 3.4 ng/mL, (7) 0.34 ng/mL, and (8) 0 ng/mL. (**b**) Sigmoidal curve of the corrected brightness of HBsAg over a concentration range from 3.4 × 10^2^ pg/mL to 3.4 × 10^8^  pg/mL. The upper inset is the schematic of the colorimetric ELISA for detection of HBsAg, where a primary antibody (rabbit anti-HBsAg) and an ALP-conjugated secondary antibody (goat anti-rabbit IgG) are used together to form a sandwich-type immunoassay. The lower inset shows the calibration curve of HBsAg where the range of linearity lies between 0.34 ng/mL to 3.4 × 10^4^  ng/mL.

Slightly different from the IgG detection, the ELISA for the detection of HBsAg was based on a sandwich-type immunoassay. As illustrated in the inset of Fig. 5b, the antigen HBsAg was first immobilized on the paper surface in the hybrid microfluidic microplate, followed with reactions with the primary antibody (i.e. rabbit anti-HBsAg) and the secondary antibody goat anti-rabbit IgG conjugated with ALP. After the formation of the sandwich structure, the enzymatic reaction between ALP and the colorimetric substrate BCIP/NBT produces the purple colour, similar to IgG detection. Different concentrations of HBsAg ranging from, 0.34 ng/mL to 340 μg/mL were analyzed by the hybrid microfluidic microplate. Figure 5a shows a scanned image for HBsAg detection from an office scanner. The purple colour intensity increased with increasing concentrations from 0.34 ng/mL to 340 μg/mL (from right to left) with the blank in the rightmost column. Figure 5b shows the calibration curve of HBsAg over a concentration range from 3.4 × 10^2^ pg/mL to 3.4 × 10^8^  pg/mL. A sigmoidal curve (Fig. 5b) was observed over the whole detected concentration range as in IgG. In case of HBsAg, the range of linearity was observed between 3.4 × 10^2^ pg/mL to 3.4 × 10^7^  pg/mL (inset Fig. 5b) with a regression curve of y = 17.37 log (x) + 56.71 (r^2^ = 0.99). The LOD for HBsAg was found to be 1.3 ng/mL, comparable to commercial ELISA kits^51^.

### Rapid Quantitative Detection of HBsAg in human serum samples

For the validation of analytical accuracy and to determine its feasibility for detection of real human samples, normal human serum was spiked with different concentrations of standard HBsAg. Four different concentrations of HBsAg (3.4 ng/mL, 34 ng/mL, 0.34 μg/mL, and 3.4 μg/mL) within the range of linearity and above the LOD were chosen for spiking and recovery tests. As can be seen from Supplementary Table S1, the intensity of purple colour increased from lower concentrations to higher concentrations of the spiked human serum samples, consistent with ELISA results using standard HBsAg (Fig. 5a). The analytical recoveries of the serum samples ranging from 91.1–109.1% were obtained and were within the acceptable criteria for bio-analytical validation^52^,^53^.

## Discussion

We have developed a simple, portable, and POC paper/PMMA hybrid microfluidic microplate for rapid and sensitive detection of infectious diseases and other bio-analytes. To the best of our knowledge, this is the first report of a paper/PMMA hybrid microfluidic device, which draws more benefits from both substrates. The innovative use of 3D micro-porous paper in funnel-shaped microwells of this hybrid microplate facilitated rapid immobilization of antibody/antigen and also avoided complicated surface modifications. ELISA assays can be completed within one hour, and results can be observed by the naked eye or scanned by an office scanner for quantitative analysis. In addition, smartphone cameras can also be used to capture the image and the signals can be processed using different applications or cloud-based systems^54^. Although the basic system shown here can only perform 8 seven-repeated experiments (7 × 8 microwells), the design can be simply modified to perform as many experiments and repeats as desired. For instance, the hybrid microfluidic microplate can be expanded to 96 wells or 384 wells according to different needs simply by increasing the number of wells and channels, while the basic architecture remains the same. Without using any specialized laboratory equipment, the LOD of 1.6 ng/mL for IgG was achieved, which is comparable to that of commercial ELISA kits using spectrometers or microplate readers. The hybrid microfluidic microplate significantly reduces the sample and reagent volume compared to commercial ELISA and shows great promise as a POC device for rapid, sensitive and quantitative detection of biomarkers, especially in low-resource settings, such as small clinics, rural areas, border regions and developing nations. Because ELISA and microplates are widely used, this hybrid paper/PMMA microfluidic microplate will have broad applications from biology and clinical diagnosis to various biochemical analyses.

## Methods

### Microfluidic platform fabrication

The chip used in this study was designed by using Adobe Illustrator CS5 and micro-machined using laser cutter (Epilog Zing 16, Golden, CO). In the mask-less laser ablation, the PMMA substrate was placed on a stage and the focused laser beam was moved across in x and y directions as defined in the designed pattern. Pieces of chromatography paper were cut using a laser cutter and placed inside each microwells, as a 3D surface for ELISA. Chromatography paper can also be placed just over the middle layer, so that the paper pieces directly fall to each microwells in the middle layer during laser cutting so that there is no need to place the chromatography paper manually to all the wells.

To assemble the device, different PMMA layers were clamped together and kept in an oven at 115–120 °C for 35 minutes. The chip could be used once it cooled down to room temperature. Different PMMA layers could be separated after an assay by applying slight pressure between the joints so that the device can be reused after cleaning.

### IgG and HBsAg detection using the hybrid device

**Figure 6:**
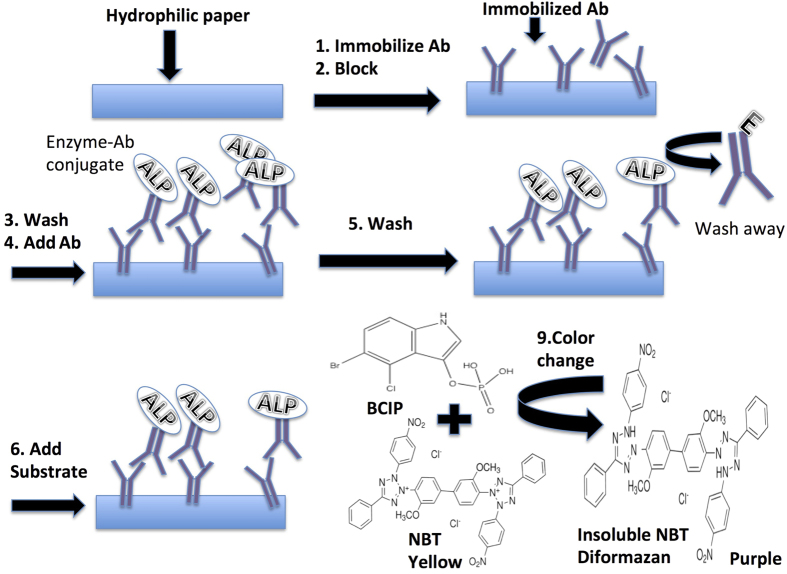
Schematic illustration of the approach of enzymatic immunoassay on the paper/PMMA hybrid device, comprising six steps: (1) Immobilizing antibody on paper, (2) Blocking, (3) Washing, (4) Binding of enzyme conjugated antibody, (5) Washing, and (6) Enzymatic production of insoluble NBT diformazan.

The hybrid device can be used for a wide range of bioassays. Figure 6 illustrates the main steps for the IgG detection by on-chip ELISA using the hybrid device. The primary antibody IgG (0.1 ng/mL-100 μg/mL in 10 mM, pH 8.0 PBS (Phosphate-buffered saline)) was introduced to the chip from different inlet reservoirs in the first layer of the chip. After the chip was incubated with the primary antibody for 10 minutes, the unreacted paper surface was blocked with a blocking buffer (4% BSA w/v in PBS + 0.05% Tween 20) for another 10 minutes. After washing with PBST (10 mM, pH 7.4 PBS + 0.05% Tween 20), anti-rabbit IgG-alkaline phosphatase (6 μg/mL) was added for another 7 min. Then, the final wash was done with the washing buffer for three times. Finally, the substrate for the alkaline phosphatase, i.e., BCIP/NBT (Nitroblue tetrazolium + 5-bromo, 4-chloro, 3-indoyl phosphate) was added. NBT is often used with the alkaline phosphatase substrate BCIP in western blotting and immunohistological staining and immunoassay procedures. These substrate systems produce an insoluble NBT diformazan, which changes the colour of the solution from light yellow to purple and can be observed visually. After 10-minute incubation, different layers of chip were separated and the middle layer was scanned with a scanner after another 15 minutes.

Regarding HBsAg detection, a similar assay procedure was followed. The main difference was that the first step was to immobilize the antigen, i.e., HBsAg, followed by addition of anti-HBsAg, and finally forming a sandwich-structure immunoassay by the addition of ALP-labelled anti-rabbit IgG. 35 μL of sample/reagents was used for each channel of the microfluidic platform. 35 μL of the reagent added to each inlet microwells, travels from the upstream delivery channel to the downstream waste channel through the different microwells (7 microwells in each channel). Hence, the average volume per well of this platform is (35/7) 5 μL per microwell.

## Additional Information

**How to cite this article**: Sanjay, S. T. *et al*. A paper/polymer hybrid microfluidic microplate for rapid quantitative detection of multiple disease biomarkers. *Sci. Rep.* **6**, 30474; doi: 10.1038/srep30474 (2016).

## Supplementary Material

Supplementary Information

## Figures and Tables

**Table 1:** Comparisons among different ELISA devices.

Device	Volume/well (channel)	Total time	LOD	POC detection	Remarks	Reagent addition	References
96 well plate ELISA	50–100 μL	Overnight + 6.5 hr.	1.6–6.25 ng/mL	☒	Spectrophotometer required	Manual/Robotic pipetters	45
Paper-based ELISA	3 μL	1 hr	54 fmol/zone	☒	Scanner required	Manual	28
PMMA device	30 μL	12 + 1hr	0.12 μg/mL	☒	Modified with APTES, fluorescence microscopy required	Syringe pump	34
Our device	5 μL	1 hr	1.6 ng/mL	☑	Desktop scanner	Automatic	
