# Supplementary material for: A paper/polymer hybrid microfluidic microplate for rapid quantitative detection of multiple disease biomarkers
Source: Sci Rep. 2016 Jul 26;6:30474. doi: 10.1038/srep30474 (PMC4960536; doi:10.1038/srep30474)
Supplement: Supplementary Information [file srep30474-s1.pdf]

Supplementary Information

A paper/polymer hybrid microfluidic microplate for rapid quantitative detection of multiple disease biomarkers

Sharma T. Sanjay,[1] Maowei Dou,[1] Jianjun Sun,[2] and XiuJun Li[1,2,3]*

[1]*Department of Chemistry, University of Texas at El Paso, 500 West University Ave, El Paso, Texas, 79968, USA.*

[2]*Border Biomedical Research Center, University of Texas at El Paso, 500 West University Ave, El Paso, Texas, 79968, USA.*

[3]*Biomedical Engineering, University of Texas at El Paso, 500 West University Ave, El Paso, Texas, 79968, USA.*

*Corresponding author: xli4@utep.edu

Table of Contents

EXPERIMENTAL SECTION…....…………………………….........................3

Chemicals and Materials …………………………………………….......….......3

Data Analysis...……………………........….3

Detection of HBsAg in human serum……………………………..……………...4

RESULTS AND DISCUSSION…………………….....................................4

Two-level laser fabrication of PMMA……...…............4

Laser ablation of PMMA (Figure S1)………..…....6

Cross contamination/leakage test (Figure S2)………...................……................7

Detection of HBsAg in human serum sample (Table 1).......................................8

References ………………………………………………………………………9

MATERIALS AND METHODS

Chemicals and Materials:

ELISA. IgG from rabbit serum, anti-rabbit IgG-alkaline phosphatase, BCIP/NBT liquid substrate, Tween 20, albumin from bovine serum, serum from normal human male AB plasma, and Phosphate Buffer Saline were purchased from Sigma Aldrich (St. Louis, MO). HBsAg protein (subtype ad) was purchased from Fitzgerald Industries International Inc., Acton, MA. Polyclonal anti-HBsAg was purchased from Novus Biologicals, Littleton, CO. Cy3-labeled IgG whole molecule was purchased from Jackson Immunoresearch Laboratories Inc., West Grove, PA, to confirm rapid IgG immobilization on paper.

Microfluidic platform fabrication. PMMA was purchased from McMaster-Carr, Los Angeles, CA. Whatman #1 chromatography paper was purchased from Sigma Aldrich (St. Louis, MO). Unless otherwise noted, all solutions were prepared with ultrapure Milli-Q water (18.2 MΩ.cm) from a Millipore Milli-Q system (Bedford, MA).

Data analysis. Once the chip was scanned using a portable flatbed scanner (CanoScan LiDE 700F, Canon), average brightness of each test zone was measured using ImageJ software, distributed for free by NIH (http://rsb.info.nih.gov/ij/download.html). The signal of an individual microwell was calculated as the average of the intensity values of respective pixels. RGB images can be converted to the gray scale using the formula gray = (red + green + blue) ⁄ 3. The display range in ImageJ from minimum to maximum is

scaled from 0 to 255. After the average brightness value was measured using ImageJ, it was subtracted from the maximum value i.e., 255 to get the corrected brightness value, which was then used for data analysis. The value obtained with concentration 0 pg/mL in PBS was defined as background.

Rapid detection of HBsAg in human serum: To validate the reliability of the developed hybrid microfluidic microplate and to test its feasibility for real human sample detection, HBsAg was spiked in normal human serum. 10 μL of varying concentrations of HBsAg were spiked into 1.0 mL human serum which was pre-diluted 3 folds using PBS to get the final concentrations of 3.4 ng/mL, 34 ng/mL, 0.34 μg/mL, and 3.4 μg/mL, respectively. After mixing thoroughly the spiked samples were used for the rapid detection of HBsAg, following steps similar to the detection of standard HBsAg prepared in PBS buffer. Spike recoveries were calculated and listed in Supplementary Table S1. Blood serum can be separated by centrifuging at 1,600 g for 15 min following 30 min of clotting at room temperature.[1] The resulting supernatant (serum) can be diluted 3 times using PBS and used as the sample for ELISA. Microfluidic methods of sample preparations designed to aid point-of-care (POC) diagnosis can be found in several articles[2,3] for separation of serum from blood. In addition, commercially available devices including seraSTAT®, Vivid™, and blood separation membrane (a product of Membrane Technologies), can separate blood within 3 minutes and can be used in POC settings. Especially, blood separation membrane can be integrated on the microfluidic device for POC analysis of real patient samples.

RESULTS AND DISCUSSION

Two-level laser fabrication of PMMA. Since the power and the speed of the laser cutter can affect the depth of microstructures, in order to achieve the desired depth, we first systematically investigated the relationship between the depth, power, and the speed of the laser cutter, as shown in Supplementary **Fig. S1.** It can be seen that with the increase of the power for a 30 W CO_2 laser in the raster mode, the structure depth increases correspondingly. Additionally, the laser cutter speed also affects the fabrication depth. The faster the laser's speed, the shallower is the depth of microstructures. Thus, the percentage power and the speed required for cutting PMMA was empirically determined. As seen from Supplementary **Fig. S1,** the speed of 10% and power of 50% could completely pass through a 2 mm thick PMMA layer creating a reservoir. Similarly, for 1.5 mm thick PMMA, speed of 10% and power of 35% could be used. Based on the data from Supplementary **Fig. S1,** an upper microwell 0.7 mm in depth was created in 2 mm thick PMMA using speed of 30% and power of 40%. Subsequently before moving the PMMA substrate, 1.3 mm deep lower microwell was created, just below the upper microwell, using speed of 30% and power of 75%.

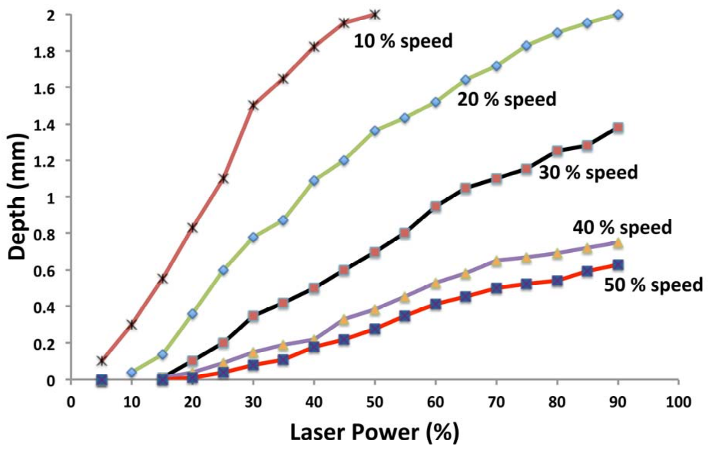

Supplementary Figure S1: Laser ablation of PMMA at different speeds and powers for two-level microfabrication of funnel-shaped microwells. The graph shows the depth of microwells achieved by speed of 10%, 20%, 30%, 40%, and 50%, respectively at different power percentages of a 30 W laser in the raster mode.

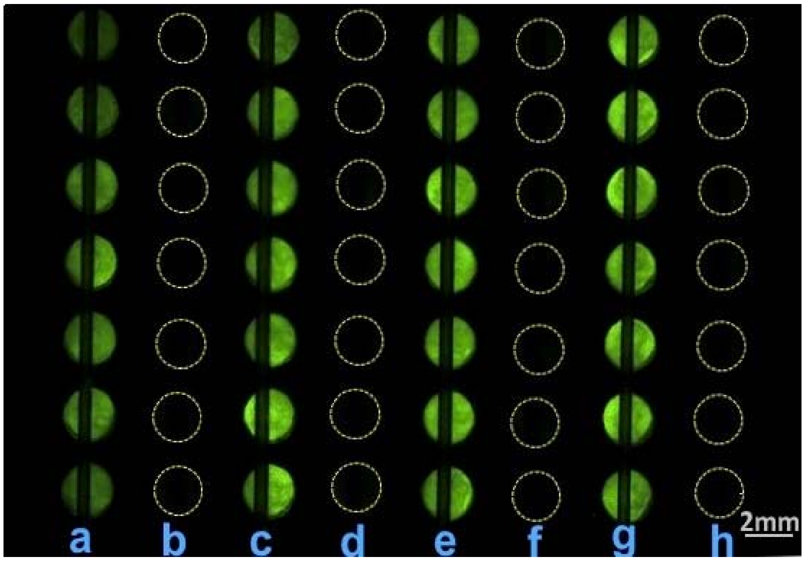

Supplementary Figure S2: Cross contamination/leakage test for the hybrid chip. Fluorescence was observed in columns a, c, e, and g where FITC was added, but not in columns b, d, f, and h, where Milli-Q water was added.

Supplementary Table S1: Detection of HBsAg spiked in human serum sample by colorimetric ELISA on a hybrid microfluidic microplate.

**Serum Sample Number**	**Serum HBsAg concentration**	**Color changes**	**Measured Values**	**Recovery (%)**
Control	0			
No.1	3.4 ng/mL	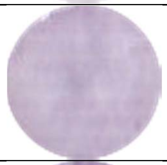	3.71 ng/mL	109.1
No.2	34 ng/mL	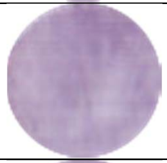	36.45 ng/mL	107.2
No.3	0.34 μg/mL	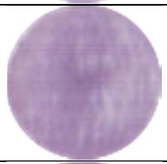	0.31 μg/mL	91.1
No.4	3.4 μg/mL	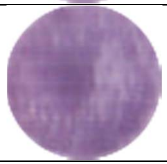	3.17 μg/mL	93.2

REFERENCES

1. Geekiyanage, Hirosha, et al. "Blood serum miRNA: non-invasive biomarkers for Alzheimer's disease." *Exp. Neurol.* 235.2 (2012): 491-496.
2. Cui, Francis, et al. "Microfluidic Sample Preparation for Medical Diagnostics." *Annu Rev Biomed Eng.* 17 (2015): 267-286.
3. Liu, Changchun, et al. "Membrane-based, sedimentation-assisted plasma separator for point-of-care applications." *Anal Chem.* 85.21 (2013): 10463-10470.
